# Hopelessness for family members of individuals with borderline personality disorder

**DOI:** 10.1111/famp.13045

**Published:** 2024-09-05

**Authors:** Mary Joyce, Mary Kells, Emily Boylan, Paul Corcoran, Bláthín Power, Stephanie Wall, Daniel Flynn

**Affiliations:** ^1^ National DBT Training Team, Health Service Executive Cork Ireland; ^2^ National Suicide Research Foundation Cork Ireland; ^3^ Mental Health Services Cork Kerry Community Healthcare, Health Service Executive Cork Ireland; ^4^ School of Public Health, University College Cork Cork Ireland

**Keywords:** borderline personality disorder, family connections, family members, hopelessness, significant others

## Abstract

Family members and loved ones of individuals with Borderline Personality Disorder (BPD) can experience high levels of distress. Types of distress reported by family members include burden, grief, depression, guilt, and powerlessness. Hopelessness is a construct that has received little attention despite its potential relevance for this group. This study sought to examine, and assess potential change in, hopelessness among individuals attending a 12‐week Family Connections (FC) program. Participants were 75 family members, 29 men and 46 women. Most participants were parents (*n* = 43; 57%). Data were collected at four time‐points and outcomes included hopelessness, burden, and grief. The majority of participants (82%) reported scores within the ‘minimal’ or ‘mild’ ranges of hopelessness before the FC program. A greater proportion of participants in the 60–70 year age group reported scores in the ‘moderate/severe’ category when compared with younger age groups. The mean hopelessness score for all participants before FC was 4.61 which is considered mild. There was no significant difference in hopelessness scores after program completion. Although mean scores increased at both 3‐month and 12‐month follow‐ups, they continued to remain in the ‘mild’ category. Hopelessness scores in the current study are similar to those reported in previous studies, although no significant change was found after FC completion. Concepts of personal vs. situational hopelessness should be considered, as well as the relevance of assessing personal hopelessness for this participant group. Further research is needed to determine the relationship between family member hopelessness and index client wellbeing.

Borderline Personality Disorder (BPD) is a mental health diagnosis characterized by a pervasive pattern of instability in interpersonal relationships, self‐image, affect, and marked impulsivity (American Psychiatric Association, 2013). BPD typically features patterns of cognitive, emotional, and behavioral dysregulation that often manifest in self‐harm and suicidal behavior (Koerner et al., 2020; Kuo et al., 2006). BPD is challenging both for individuals who experience these difficulties and for family members/significant others. Family members of individuals with BPD commonly report feeling overwhelmed and traumatized by the behaviors that frequently accompany BPD such as suicide attempts, intense anger, and self‐injury, which result in highly stressful and chaotic situations for the person with BPD and their families (Hoffman et al., 2007).

Previous research has found that family members of individuals with BPD experience higher levels of psychological and somatic distress than the general population (Scheirs & Bok, 2007). Carers of young people with BPD features have reported elevated levels of distress and higher levels of negative experiences related to their caregiving role when compared with general population adults or carers of young people with other serious illnesses such as eating disorders or psychosis (Cotton et al., 2022; Seigerman et al., 2020). Types of distress reported by family members include burden, grief, depression, guilt, and powerlessness (Bailey & Grenyer, 2014; Ekdahl et al., 2011; Hoffman et al., 2005). A systematic review which examined the experience of carers of individuals with personality disorder reported elevated burden, grief, impaired empowerment, and mental health problems including depression and anxiety (Bailey & Grenyer, 2013). Five out of the six studies in that review focused on carers of individuals with BPD, making the findings particularly relevant to the current study.

Hopelessness has also been identified as a potentially relevant construct for family members of individuals with BPD. The distinctive characteristics of BPD, such as intense emotion dysregulation and interpersonal conflict, provide a basis for understanding why caregivers of individuals with BPD may experience heightened levels of hopelessness. Hopelessness was first examined in this population in a study by Hoffman and colleagues whereby the relationship between family members' knowledge about BPD and levels of burden, depression, and hopelessness was assessed (Hoffman et al., 2003). It was found that more knowledgeable family members reported greater feelings of hopelessness, though overall as a group, participants reported a mean score within the ‘mild’ category of severity on the Beck Hopelessness Scale (BHS). A later qualitative study with family members found that prolonged hopelessness was one of five key areas identified as central to life with their relative (Buteau et al., 2008). Multiple failed attempts at treatment, unsatisfactory support systems, and both emotional and financial exhaustion were noted as reasons for family members' despair that their life and the lives of their ill relative may never improve (Buteau et al., 2008). The possibility that hopelessness could be a relevant construct for this population was also raised in an interventional study by our research group when anecdotal evidence from family members led us to consider that hopelessness may be a relevant construct worthy of further investigation (Flynn et al., 2017). It was also found that depression scores among family members in that study were lower than those reported in earlier similar studies conducted by Hoffman et al. (2005, 2007). We hypothesized that cultural variance in attitudes towards depression in an Irish context may have influenced individuals' responses, and that hopelessness might be a more meaningful construct to investigate in this population, as well as being a potentially more socially acceptable one to endorse. Most recently, a phenomenological study examining the experiences of caregivers of individuals diagnosed with BPD found that emotional distress including hopelessness impacted various aspects of the caregiver's life and functioning including their physical health and ability to focus on work (Meshkinyazd et al., 2020). Participants also expressed concerns about the negative stigmatization in society associated with a BPD diagnosis, leading to further feelings of shame and hopelessness.

Despite its potential relevance, hopelessness has received little attention in studies involving family members of individuals with BPD. A recent systematic review of interventions for family members and carers of individuals with BPD (Guillén et al., 2020) lists hopelessness as an outcome measure in only one of 11 studies. That study, by Miller and Skerven (2017), evaluated a family‐oriented Dialectical Behavior Therapy (DBT) program called Family Skills, in which participants reported a mean hopelessness score within the ‘mild’ category of severity at pre‐treatment. The majority of participants (63%) reported scores within the lowest category of severity (‘minimal’). A reduction in hopelessness scores was reported at post‐treatment and there was an increase in the proportion of participants scoring within the ‘minimal’ category (72%). Clinical significance of the statistical change was investigated but most participants were unclassifiable due to having scores within what the authors describe as a ‘functional’ range at pre‐treatment.

While these studies demonstrate the impact that caring for a loved one with BPD can have on family members, there is a lack of support programs available for this group. This contrasts with family programs developed for relatives of individuals with psychiatric disorders other than BPD, which have taken a valued role in treatment settings (Hoffman et al., 2005). Family Connections (FC) was the first education and support program to be designed specifically for family members who have a relative with BPD (Hoffman et al., 2007). FC is a 12‐week manualized skills training and support program designed to meet the needs of family members by providing: (a) education about BPD and family functioning, (b) individual and family skills to help with managing one's own negative reactions, and building better and more satisfying relationships, and (c) social support from other group members who have lived through similar experiences and are living with similar scenarios (Hoffman & Fruzzetti, 2007). FC is the most researched program for family members of individuals with BPD (Guillén et al., 2020, 2022) with studies reporting reductions in burden, grief, and depression, and increases in personal mastery (Flynn et al., 2017).

While FC does not explicitly target hopelessness, there is a clinical rationale for anticipating that each of the three foci of FC would decrease hopelessness. Through the provision of up‐to‐date and accurate psychoeducation about BPD, it is likely that inaccurate myths about the prognosis of BPD would be dispelled. This may help to instil hope of recovery among the family members' relative with BPD. FC also teaches family members mini DBT skills. In the practical application of these skills, family members may then be able to change the course of interpersonal interactions with their loved one with BPD, thus moving the relationship transactions in a healthier direction. As healthier transactions develop, family member hopelessness may decrease. Finally, FC attempts to foster support among participants. The unique support which participants frequently report they receive through FC fosters a sense of authentic validation. This validation from other participants can help family members to become more emotionally regulated and subsequently improve their own capacity for interpersonal problem‐solving. This can set the context for family members becoming more hopeful.

As there has been minimal research on hopelessness in family members of individuals with BPD, and no FC study that has included a measure of hopelessness, we sought to examine levels of hopelessness among individuals attending a FC program. We also aimed to assess potential change in hopelessness scores from pre‐ to post‐FC, as well as at two follow‐up time‐points up to 12‐month post program completion.

## METHOD

### Design and study setting

This study takes the form of a quasi‐experimental design without randomization or a control group. Data were collected from six cohorts of a FC program delivered in a community mental health service in Ireland between February 2014 and January 2019. All FC programs were delivered in‐person. Family members had to be 18 years or older to participate in FC. Attendees were primarily nominated by their relative/significant other with BPD (the index client) who was engaged with the community mental health service within which the study was conducted. One or more family members could be nominated for the FC program. The FC programs were facilitated by two clinician leaders trained in FC, and where feasible, a family member who had completed FC Leader Training as well.

### Participants

Participants recruited for this study were attendees at the six cohorts of the FC program in Cork, Ireland during the timeframe outlined above. They were a family member, or a significant other, of individuals with severe emotion and behavior dysregulation who may have received a diagnosis of BPD (for clarity, the family member experiencing emotion and behavior dysregulation will be referred to as the index client). A total of 75 participants consented to participate, ranging in age from 22 to 70 years, with more than half aged 40–59 years. They are comprised of family members (parents, children, or siblings) and other significant people in their lives (spouses, partners, or friends). The majority of participants (57%) were parents. Demographic information of participants is provided in Table 1.

**TABLE 1:** Sociodemographic characteristics of participants.

Baseline characteristic	Full sample (*n* = 75)	
	*n*	%
**Sex**		
Men	29	39
Women	46	61
**Age group**		
20–29 years	7	9
30–39 years	11	15
40–49 years	20	27
50–59 years	24	32
60–70 years	13	17
**Relationship to index client**		
Parent	43	57
Spouse/partner	18	24
Sibling/child/friend	12	16
Unknown	2	3

### Measures

The measures used in this study were the Beck Hopelessness Scale, the Burden Assessment Scale, the Grief Assessment Scale, the Personal Mastery Scale, and the Revised Centre of Epidemiologic Studies Depression Scale. All are self‐report questionnaires. They are in line with previous research on FC (e.g., Hoffman et al., 2005, 2007), with the addition of the Beck Hopelessness Scale. Outcome measures mostly matched those utilized in previous FC studies to allow for comparison across studies. The listed measures (except for the BHS) were originally selected by the FC developers as they were commonly used measures in the schizophrenia caregiver research (Hoffman et al., 2007). This consistency has allowed comparisons across caregiver groups. The current study added a measure of hopelessness, prompted by the anecdotal information about the relevance of this construct noted by Flynn et al. (2017).

The Beck Hopelessness Scale (BHS; Beck & Steer, 1988) is a 20‐item scale that measures participant's levels of hopelessness. Scores range from 0 to 20 and are categorized according to four levels of severity (Minimal: 0–3; Mild: 4–8; Moderate: 9–14; and Severe: 15–20). The scale demonstrated good internal reliability in this study (*α* = 0.91).

The Burden Assessment Scale (BAS; Reinhard et al., 1994) was used to measure the construct of burden, which measures subjective and objective burden. The internal reliability of the BAS subscales in this study was 0.89 for objective burden and 0.86 for subjective burden.

The Grief Assessment Scale (GAS; Struening et al., 1995) is a 15‐item scale that measures an individual's current feelings of grief. The scale demonstrated good internal reliability (*α* = 0.94).

The Personal Mastery Scale (PMS; Pearlin et al., 1981) is a 7‐item scale that measures participants perceived level of coping. The scale demonstrated good internal reliability in the current study (*α* = 0.76).

The Revised Centre of Epidemiologic Studies Depression Scale (CES‐D; Radloff, 1977) is a 20‐item scale that measures individuals' levels of depressive symptoms experienced. The scale demonstrated good internal reliability in this study (*α* = 0.90).

Participants were also asked the following questions about their relative/ significant other (index client): the frequency of contact on a weekly basis; the timeframe of their BPD diagnosis (if applicable); what kind of treatment, if any, the index client was receiving; and the length of time the index client had been receiving treatment.

### Procedure

All individuals who participated in FC programs in Cork, Ireland between February 2014 and January 2019 were invited to participate in this study. Individuals were invited to participate in the research study either prior to or during the first session of the FC program. Potential participants were briefed on the details of the study and provided with information leaflets. Written informed consent was obtained for those who opted into the study. Participants completed the pre‐program questionnaires during the first session of the FC program or within the first 2 weeks of the program start date if completing the questionnaires at home. At time‐point 2, participants completed the questionnaires in the final session of FC or within 2 weeks of program completion. There were two follow‐up data collection time‐points for participants who completed FC: 3 months and 12 months after program completion. At both follow‐up time‐points, participants were contacted by telephone. Where participants were willing to continue to participate, address details were obtained so that the questionnaires could be sent via post for completion at home. Participants were asked to return the completed questionnaires within a week of receiving them using the prepaid envelope provided.

### Data analysis

Descriptive statistics were used to summarize the data at each time‐point and outcome measures were summarized by their mean and standard deviation. *T*‐tests and analyses of variance were used to assess sex, age and relationship type differences in the baseline outcome measures. Linear mixed‐effects models were used to assess differences in the outcome measures from pre‐ to post‐FC. Linear mixed‐effects models utilizes all available data at each time‐point, irrespective of missing data for participants at different time‐points. Pearson product‐moment correlation coefficients were calculated to examine the relationship between constructs. IBM SPSS Versions 27 and 28 and Stata Version 15 for Windows were used to analyze the data.

### Ethics approval and consent to participate

All procedures were reviewed and approved by the Clinical Research Ethics Committee of the Cork Teaching Hospitals. Written informed consent was obtained from all participants.

## RESULTS

**FIGURE 1:**
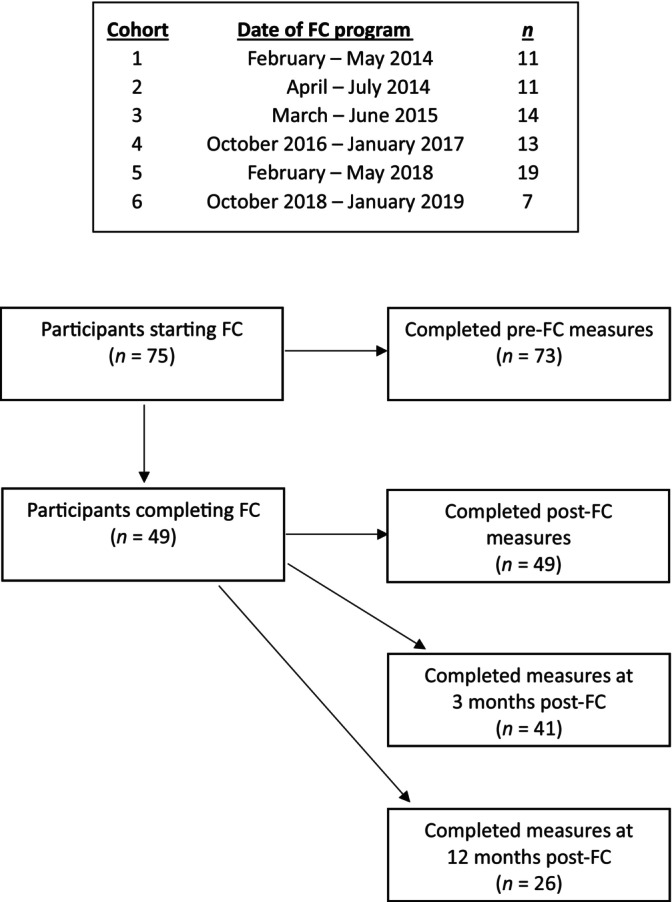
Timeline of FC programs and number of participants per cohort, and flowchart of participants.

Figure 1 provides the timeline of FC programs and number of participants in each cohort, as well as the flowchart of participants. Forty‐nine of 75 participants completed FC. The program drop‐out rate was 35%.^1^ Reasons for drop‐out included work schedule conflicts, family commitments such as childcare, and participants' family member (index client) disengaging from their treatment program. On some occasions, particularly when drop‐out happened early in the program, the reason for drop‐out was unknown.

Of the 75 participants who took part in this study, 73 participants completed questionnaires at time‐point 1. There were incomplete or ambiguous answers on the BHS for a further two participants resulting in available data for 71 participants at time‐point 1. Descriptive statistics for the BHS for those 71 participants are presented in Table 2.

**TABLE 2:** Descriptive statistics for the BHS and levels of severity for all participants pre‐FC.

Descriptive statistic	Pre‐FC		
		*n*	%
Mean	4.61		
SD	4.82		
Range	0–18		
**Level of severity**			
Minimal (0–3)		41	58
Mild (4–8)		17	24
Moderate (9–14)		8	11
Severe (15–20)		5	7

*Note*: Data for the BHS were available for 71 of 75 participants at time‐point 1 (pre‐FC).

The mean score for all participants at the beginning of FC was 4.61 which is in the ‘mild’ category. Most participants (82%) scored in the ‘minimal’ or ‘mild’ categories of hopelessness prior to FC.

Descriptive statistics for hopelessness scores according to demographic variables and other potential related factors such as timeline of the index client's diagnosis are outlined in Table 3. The proportion of participants reporting hopelessness scores in the ‘moderate/severe’ category at pre‐FC was similar for men and women. A greater proportion of those in the 60–70 year group reported scores in the ‘moderate/severe’ category when compared with other age groups. In terms of the type of relationship to the index client, only parents and spouses/partners reported scores in the ‘moderate/severe’ category.

**TABLE 3:** Descriptive statistics for the BHS according to demographic variables and other potential related factors pre‐FC.

Variable	Hopelessness severity level					
	Minimal/mild (0–8)		Moderate/severe (9–20)			
	*n*	%	*n*	%	Mean	SD
**Sex**						
Men (*n* = 28)	23	82.1	5	17.9	4.48	4.38
Women (*n* = 43)	35	81.4	8	18.6	4.70	5.13
**Age group**						
20–29 years (*n* = 7)	7	100.0	0	0	3.43	2.44
30–39 years (*n* = 11)	8	72.7	3	27.3	6.55	4.70
40–49 years (*n* = 20)	17	85.0	3	15.0	3.10	3.95
50–59 years (*n* = 21)	19	90.5	2	9.5	3.71	4.90
60–70 years (*n* = 12)	7	58.3	5	41.7	7.61	5.82
**Relationship to index client**						
Parent (*n* = 41)	32	78.0	9	22.0	4.74	5.42
Spouse/partner (*n* = 18)	14	77.8	4	22.2	5.50	4.49
Sibling/child/friend (*n* = 12)	12	100.0	0	0	2.83	2.25
**Frequency of contact with index client**						
Every day (*n* = 55)	43	78.2	12	21.8	5.09	5.15
4–6 times per week (*n* = 9)	8	88.9	1	11.1	3.92	3.78
1–3 times per week or less (*n* = 7)	7	100.0	0	0	1.71	0.84
**Timeline of index client's diagnosis** ^a^						
<1 year (*n* = 15)	12	80.0	3	20.0	4.67	3.96
1–2 years (*n* = 10)	8	80.0	2	20.0	4.20	5.80
2–6 years (*n* = 6)	5	83.3	1	16.7	4.17	4.54
Don't know (*n* = 5)	4	80.0	1	20.0	6.20	6.14
**Index client receiving treatment** ^a^						
Yes; DBT (*n* = 21)	16	76.2	5	23.8	4.95	5.56
Yes; Other (*n* = 6)	6	100.0	0	0	3.33	1.75
On a waitlist (*n* = 7)	5	71.4	2	28.6	4.71	4.89
Don't know (*n* = 1)	1	100.0	0	0	6.00	N/A
**Length of time index client in treatment** ^b^						
<6 months (*n* = 21)	18	85.7	3	14.3	4.19	3.23
6–12 months (*n* = 7)	4	57.1	3	42.9	7.00	6.76
>1 yr (*n* = 3)	3	100.0	0	0	1.67	0.58
On a waitlist (*n* = 7)	5	71.4	2	28.6	4.71	4.89
Completed treatment (*n* = 5)	4	80.0	1	20.0	4.80	7.56
Other/unknown (*n* = 4)	4	100.0	0	0	3.00	2.00

^a^ Information on the timeline of the index client's diagnosis and what kind of treatment they received was recorded from October 2016 onwards (*n* = 36).

^b^ Information on length of time the index client was in treatment was recorded from March 2015 onwards (*n* = 50).

Of the 49 participants who completed FC, data were available at both pre‐ and post‐FC program for 46. As there was variation in hopelessness scores within the sample at pre‐FC, and scores from participants who dropped out could alter results, descriptive statistics for participants who completed FC only are provided in Table 4. For participants who completed FC, the mean hopelessness score at the beginning of FC was 4.85 which is in the ‘mild’ category. The mean score at post‐FC reduced to 4.58 but remained within the same category of severity. Most participants (78%) reported scores in the ‘minimal’ or ‘mild’ categories of hopelessness at the beginning of FC. This was similar at the end of the FC program (76%). Although the number of participants reporting scores in the ‘severe’ category at pre‐FC was low (*n* = 4), this reduced to two participants at post‐FC. Data were available for 34 participants at 3‐month follow‐up and 22 participants at 12‐month follow‐up. Although mean scores increased at both 3‐month and 12‐month follow‐up, they continued to remain in the ‘mild’ category. The proportion of participants reporting scores in the ‘moderate’ and ‘severe’ categories of hopelessness was largest at 12‐month follow‐up.

**TABLE 4:** Descriptive statistics for the BHS at pre‐ and post‐FC, and two follow‐ups, for participants who completed FC.

Descriptive statistic	Time‐point 1			Time‐point 2			Time‐point 3			Time‐point 4		
	*n*			*n*			*n*			*n*		
	46			46			34			22		
	*M*	SD	Range	*M*	SD	Range	*M*	SD	Range	*M*	SD	Range
	4.85	5.27	0–18	4.58	4.96	0–18	4.94	5.31	0–19	6.18	6.09	0–19
	*n*	%		*n*	%		*n*	%		*n*	%	
**Hopelessness severity level**												
Minimal (0–3)	26	56		28	61		18	53		9	41	
Mild (4–8)	10	22		7	15		11	32		6	27	
Moderate (9–14)	6	13		9	20		1	3		4	18	
Severe (15–20)	4	9		2	4		4	12		3	14	

*Note*: The results presented here only include data for participants who completed the FC program (see Figure 1).

Abbreviations: *M*, Mean; SD, Standard deviation.

Across all outcome measures, data were available for 73 participants at pre‐FC and for 49 participants at time‐point 2 (post FC). The scores on each of the outcome measures at pre‐FC according to sex, age, and relationship to index client are provided in Table 5. Women reported higher scores than men on all measures except for personal mastery. *T*‐tests were conducted to explore the observed sex differences and found significantly higher levels of total burden (*t* (70) = −1.98, *p* < 0.05) and grief (*t* (71) = −2.19, *p* < 0.05) for women in comparison to men. There was a medium effect size (Cohen's *d* = 0.49 and 0.53 for burden and grief respectively). Mean scores varied across outcome measures according to age group and relationship to index client (Table 5). One‐way between‐group analyses of variance found a significant difference in personal mastery scores (*F* (4, 68) = 2.68, *p* = 0.04) and hopelessness scores (*F* (4, 66) = 2.60, *p* = 0.04) across age groups. Post‐hoc comparisons using the Tukey HSD test indicated that 60–70‐year‐olds reported significantly lower personal mastery scores (*M* = 17.77, SD = 3.44) when compared with 40–49 year olds (*M* = 21.55, SD = 3.58). The observed difference in hopelessness scores between 60–70‐year‐olds (*M* = 7.61, SD = 5.82) and 40–49‐year‐olds (*M* = 3.10, SD = 3.95) approached significance. The effect sizes were large (eta squared = 0.14). There were no differences in scores according to index client relationship.

**TABLE 5:** Means and standard deviations for each outcome measure by sex, age group, and family member type.

Baseline characteristic	Hopelessness		Objective burden		Subjective burden		Total burden		Grief		Personal mastery		Depression	
	*n*		*n*		*n*		*n*		*n*		*n*		*n*	
	71		72		72		72		73		73		71	
	M	SD	M	SD	M	SD	M	SD	M	SD	M	SD	M	SD
**Sex**														
Men	4.48	4.38	21.81	6.89	24.43	6.31	46.24	12.29	44.02	14.16	20.57	3.29	14.43	8.40
Women	4.70	5.13	25.11	7.29	27.33	6.92	52.43	13.33	51.23	13.35	19.58	4.29	16.57	10.72
**Age group**														
20–29 years	3.43	2.44	21.41	7.90	26.14	7.78	47.56	15.28	43.41	13.21	22.00	2.58	16.71	7.06
30–39 years	6.55	4.70	26	5.93	28.63	5.84	54.64	11.17	47.27	12.49	19.63	2.87	18.78	10.96
40–49 years	3.10	3.95	22.97	7.17	24.17	6.34	47.14	12.34	45.05	14.99	21.55	3.58	13.33	10.69
50–59 years	3.71	4.90	25.56	7.93	27.86	7.09	53.43	14.15	51.62	14.57	19.32	4.67	15.32	10.76
60–70 years	7.61	5.82	21.47	6.65	24.33	6.76	45.80	12.51	52.11	12.92	17.77	3.44	17.03	7.21
**Relationship to index client**														
Parent	4.74	5.42	24.07	7.67	26.01	7.17	50.08	14.07	50.8	14.24	19.33	4.28	14.94	9.65
Spouse/Partner	5.5	4.49	25.54	6.52	26.72	6.59	52.26	12.33	46.6	14.55	20.00	3.31	18.33	11.62
Sibling/Child/Friend	2.83	2.25	20.41	6.16	26.08	6.20	46.49	11.41	42.91	11.17	22.17	2.82	14.47	7.51
**Total**	4.61	4.82	23.82	7.27	26.20	6.79	50.02	13.20	48.47	14.02	19.96	3.94	15.72	9.86

Abbreviations: *M*, Mean; SD, Standard deviation.

Linear mixed‐effects models were used to estimate the mean at pre‐FC and the mean change from pre‐ to post‐FC. Mean scores were estimated as adjustments were made for sex and age. Significant differences were found from pre‐ to post‐FC for objective burden, subjective burden, total burden, grief, and depression (Table 6).

**TABLE 6:** Estimated means (*M*) and confidence intervals (CI) at pre‐and post‐FC.

Variable	Pre‐FC		Post‐FC		Cohen's *f* squared
	*M*	95% CI	*M*	95% CI	
Hopelessness	3.47	[1.66, 5.27]	3.54	[1.71, 5.37]	0.01
Objective burden	25.41	[22.65, 28.17]	21.54	[18.77, 24.32]*	0.42
Subjective burden	27.64	[24.90, 30.40]	22.53	[19.73, 25.33]*	0.49
Total burden	53.04	[47.83, 58.24]	43.98	[38.71, 49.25]*	0.34
Grief	52.61	[47.17, 58.05]	44.89	[39.44, 50.34]*	0.38
Personal mastery	19.48	[18.10, 20.86]	20.27	[18.89, 21.65]	0.06
Depression	14.88	[11.01, 18.75]	10.45	[6.48, 14.42]*	0.22

*Note*: Data presented here are estimates based on all available data at each time‐point (73 participants at pre‐FC and 49 participants at post‐FC).

* *p* < 0.001.

The relationship between the constructs examined in this study was also investigated. Pearson product–moment correlation coefficients were obtained and are presented in Table 7. There was a strong, positive correlation between hopelessness and depression with higher levels of hopelessness associated with higher levels of depression. A strong, negative correlation was found between hopelessness and personal mastery where higher levels of hopelessness were associated with lower levels of mastery. There were medium correlations between hopelessness and burden and grief.

**TABLE 7:** Intercorrelations between measures of hopelessness, burden, grief, personal mastery and depression.

Variable	Objective burden	Subjective burden	Total burden	Grief	Personal mastery	Depression
Hopelessness	0.41	0.45	0.46	0.45	−0.61	0.62
Objective burden	–	0.76	0.94	0.62	−0.38	0.39
Subjective burden	–	–	0.94	0.64	−0.41	0.43
Total burden	–	–	–	0.67	−0.42	0.43
Grief	–	–	–	–	−0.59	0.48
Personal mastery	–	–	–	–	–	−0.67

## DISCUSSION

The main aim of this study was to examine levels of hopelessness in family members attending a FC program and to assess potential change in scores from pre‐to post‐FC, and at two follow‐up time‐points. We found that most participants reported either ‘minimal’ or ‘mild’ levels of hopelessness prior to participating in FC. Participants who reported scores in the ‘moderate’ or ‘severe’ categories were either parents or spouses/partners. When examined by age group, a greater proportion of those aged 60–70 years reported scores in the ‘moderate/ severe’ category in comparison to younger age groups. For participants who completed FC, mean hopelessness scores at post‐FC were similar to that reported at pre‐FC and remained within the same category of severity (mild). Significant reductions were noted from pre‐ to post‐FC on measures of burden, grief, and depression.

This study was partially prompted by the hypothesis that a sample of family members and significant others of those with severe emotion and behavior dysregulation in an Irish health service setting, who may have received a diagnosis of BPD, may experience significant levels of hopelessness. While this hypothesis was derived from clinical observations as well as from previous studies in the area (Buteau et al., 2008; Flynn et al., 2017), it was not upheld by the data. A ‘mild’ level of hopelessness was reported at pre‐FC. It was further hypothesized that hopelessness levels may be impacted by participation in the FC program. However, participants remained within the same category of hopelessness at post‐FC and at both follow‐up time‐points. It is possible that hopelessness of family members in relation to their loved one with BPD may need to be conceptualized or measured differently. The BHS measures personal hopelessness. This is distinct from situational hopelessness. The strong correlation between hopelessness and depression in this study provides a further indication that levels of personal hopelessness are being assessed by the BHS. Perhaps family members experience hopelessness in relation to their loved one's suffering which is not captured by the BHS, and this warrants further consideration.

Sex of participants did not appear to impact hopelessness scores. However, both age and family relative type appeared to have some bearing on levels of hopelessness. Participants in older age groups tended to have higher hopelessness scores. This may be related to having a longer‐lived experience of observing their loved one suffer from chronic emotion and behavior dysregulation. In terms of family member type, parents and partners tended to have higher hopelessness scores than friends or other family members. None of the participants who were siblings, friends, or adult children of those with BPD scored above 9 on the BHS. It's possible that parents and partners are more closely and directly involved in the individual's life, and that their higher hopelessness scores may reflect the interconnectedness and reciprocity of individual functioning and family functioning. It is worth considering that with a larger sample size, these observations may have been significant.

The findings from this study are in contrast to Miller and Skerven (2017) who found their family‐oriented DBT skills‐based program led to a decrease in hopelessness levels over the course of the program. It is unclear why there was a decrease in hopelessness scores following participation in Miller and Skerven's DBT skills‐based program but not in FC. It is noteworthy though that the sample sizes in both Miller and Skerven's study and the current study were relatively small so the findings may be sensitive to large changes by individual participants. As an example of this, Miller and Skerven highlight that the magnitude of change on the BHS for two participants was notable where one participant obtained a BHS score of 19 at pretreatment and a 0 at posttreatment while the other obtained a score of 12 at pretreatment and a score of 4 at posttreatment. Large changes in scores for individual participants in a small sample may overinflate the results so caution should be exercised when interpreting the results of both studies. In the first study of hopelessness by Hoffman et al. (2003), more knowledgeable family members reported greater hopelessness. Those findings would suggest that there may be no change or indeed an increase in hopelessness scores following a support program that includes psychoeducation about BPD. It is also possible however that the significant advances in evidence‐based interventions for individuals with severe emotion and behavior dysregulation in the past 20 years since Hoffman et al.'s study have changed the direction of the relationship between knowledge about this presentation and hope.

It is plausible that many of the skills taught in FC have the capacity to impact hopelessness, either directly or indirectly. From a clinical perspective, relationship mindfulness, radical acceptance, and opposite to emotion action could be helpful to this end. When considering the lack of change in hopelessness scores from pre‐ to post‐FC, perhaps participants didn't get to a place of skills generalization where readily drawing on these skills in complex circumstances was possible. However, it is more likely that the controlling variables for hopelessness in family members lie outside of FC, and that it is closely connected with index client outcomes or other factors in their lives. Research linking family member outcomes with outcomes for the index client are necessary to illuminate this further.

It is possible that the minimal levels of hopelessness in our sample is linked with factors associated with the timing and stage of treatment of their family member. The majority of participants in this study had family members who had recently completed DBT, who were actively availing of DBT at the time, or who were on a waitlist for DBT. It is unknown if family members whose loved one is not engaged with or referred to an evidence‐based treatment might experience higher levels of hopelessness.

This study advances the understanding of hopelessness in family members of individuals with BPD. While earlier studies initially drew attention to the possible importance of this construct (e.g., Buteau et al., 2008; Hoffman et al., 2003), this was the first study specifically designed to quantitatively analyze the hopelessness construct with this group and to study its trajectory before and after a FC program and at follow‐up. Recommendations for future research which may further enhance our understanding of hopelessness for family members are outlined later.

The inclusion of outcome measures used in previous FC studies allowed us to identify if the profile of participants in this study was similar to that of participants in previous research. The results showed that participants' levels of grief and burden at baseline were similar to that reported by Hoffman et al. (2005, 2007). These findings lend evidence to suggest that the profile of participants across studies was similar.

A limitation of this study is that it was a quasi‐experimental study without randomization or a control group. Future research to further investigate hopelessness in family members should include a randomized design so the potential impact of the FC program on hopelessness scores can be further examined. It should also be noted that while the linear mixed‐effects model utilized data collected at each time‐point to examine the treatment effect on all outcome measures, the descriptive analyses conducted on the BHS were conducted only with participants who completed the program. This was done in an effort to accurately represent participants' trajectories regarding hopelessness over the course of the program. In doing so, however, the number of participants for whom comprehensive findings on the BHS were available is small.

While we descriptively explored hopelessness scores in relation to whether participants loved one was in treatment, as well as how far along in their treatment program they were, we commenced gathering this information later in our overall data collection. A limitation of this study is that we did not have sufficient power to examine the possibility that participants who may not have a loved one engaged with treatment may report higher levels of hopelessness. It will be important in future research to examine hopelessness of caregivers and the potential relationship to the degree of recovery of their loved one in treatment. However, it is still worth considering the possibility that minimal levels of hopelessness may be indicating that family members were only marginally affected by their living experiences with their loved one who experience emotion and behavior dysregulation.

A limitation of this study is not recording whether participants and their family member with BPD were living together. Although information on the frequency of contact with participants' family member with BPD was captured, Miller and Skerven (2017) noted that the experience of living with a family member with BPD may have different effects on family members depending on factors such as severity of BPD or BPD‐related behaviors, family members' resilience factors and social support. It would be important in future research to gather information on living arrangements, as well as the listed factors, to provide further contextual information for participant scores on outcome measures.

In this study, family members were typically nominated for FC by the index client with BPD. This is different from the typical international protocol for accessing FC where family members access the program via the National Education Alliance for Borderline Personality Disorder (NEABPD) and is independent of their loved one accessing mental health services. In addition, most of our sample (77%) reported daily contact with their loved one with BPD. We do not know if daily contact is related to hopelessness, but it may be useful for further research to investigate this. It would also be relevant to examine hopelessness for individuals who access FC independent of their family with BPD and to compare their outcomes with those who are nominated by their family member.

It is also possible that family members opting into a support program such as FC may already be open to the possibility of change and may be more hopeful than those who opt not to attend. Future research might explore levels of hopelessness in family members/ significant others who engage in a support program such as FC with those who decide not to.

At this point, the mechanisms of change in FC have not yet been investigated. Study of the same, in due course, will inform our thinking about the potential for FC to impact hopelessness.

Contrary to clinical observations and previous research, this study found family members/significant others of individuals with BPD reported minimal levels of hopelessness. Further research focusing on situational hopelessness needs to be conducted before any firm conclusions can be drawn in relation to levels of hopelessness in this population. Older participants, parents, and partners reported higher levels of hopelessness. Further research with larger numbers would allow a more detailed exploration of these factors. It is also recommended that future larger scale research would further address the relationship between hopelessness and demographic and treatment factors outlined.

## FUNDING INFORMATION

This research was conducted through funding provided by the Health Service Executive's National Office for Suicide Prevention.

## Footnotes

^1^
As all individuals who participated in the FC program took part in the research study, drop‐out rates for FC and the study are the same.
